# The Detransition Rate Is Unknown

**DOI:** 10.1007/s10508-023-02623-5

**Published:** 2023-06-12

**Authors:** J. Cohn

**Affiliations:** Society for Evidence-Based Gender Medicine, Twin Falls, ID 83301-5235 USA

## Introduction

The number of young people with gender dysphoria and trans identification has risen sharply in the last two decades, and the reasons for this are unknown (e.g., Aitken et al., 2015; Kaltiala et al., 2020; Zhang et al., 2021). Those with adolescent onset comprise the majority of the surge in new cases, dominated by natal females, in contrast to the much rarer earlier cases, which were dominated (~ 2:1) by prepubertal natal males. Many in this new cohort have comorbidities (Kaltiala-Heino et al., 2018); earlier cases often did as well, including anxiety (Wallien et al., 2007) and specifically separation anxiety (Zucker et al., 1996).

One treatment for young people with gender dysphoria, proposed and pioneered by a group of Dutch clinicians in the late 1990s-early 2000s (Biggs, 2023; Cohen-Kettenis & van Goozen, 1997; de Vries et al., 2014; Delemarre-van de Waal & Cohen-Kettenis, 2006), is medical intervention (i.e., puberty blockers, hormones, and/or surgeries). Hormones are often taken for one's entire lifetime and many of the medical interventions are irreversible. The current evidence for efficacy and/or safety of different aspects of medical intervention has been found in evidence reviews to be of “low” and “very low” quality or certainty (Brignardello-Petersen & Wiercioch, 2022; Hembree et al., 2017; National Institute for Health and Care Excellence [NICE] 2020a, 2020b), “insufficient” (Haupt et al., 2020, p. 2), and “insufficient and inconclusive” (Swedish National Board of Health & Welfare, 2022, p. 3). Low/very low quality (or certainty) means “the true effect may be/is likely to be substantially different from the estimate of the effect” (Balshem et al., 2011, Table 2).

Some people stop taking hormones, stop identifying as transgender, and/or regret their medical interventions. Several aspects are discussed in Jorgensen (2023). While discontinuing hormones is a clearly defined action, the terms “regret” (Kuiper & Cohen-Kettenis, 1998; Narayan et al., 2021; Pfäfflin, 1992) and “detransition” (Expósito-Campos, 2021) can refer to a variety of situations. For example, there are those who both regret and detransition, those who regret medical intervention but feel detransition is impractical given their physical changes, those who do not report regret but would have preferred, in retrospect, to not have medicalized, and those who think that their medical intervention was inappropriate; all of these outcomes provide important information. Detransition can mean no longer presenting as transgender, with or without remaining trans-identified, and may or may not involve surgeries. Detransition is not necessarily equivalent to the failure of medical intervention to resolve gender dysphoria; diverse reasons have been given by those who identify as detransitioners (Vandenbussche, 2022) and by detransitioners who still identify as transgender or non-binary (Turban et al., 2021), in convenience samples recruited online. Discontinuation can overlap with regret and/or detransition, or neither (for instance, when due to health concerns alone). The percentage of people who discontinue, detransition, and/or regret is not known, outside of some very narrowly defined study populations (Dhejne et al., 2014).

Although this fact is known to some (Irwig, 2022; Levine et al., 2022), low regret or detransition percentages are frequently quoted. Examples from the medical research literature include: “rare case of detransition,” “only 0.6% of transgender women and 0.3% of transgender men who underwent gonadectomy experienced regret” (Rosenthal, 2021, p. 586), “Post-GAC regret is rare, occurring in approximately 0.3% of individuals” (McNamara et al., 2022, p. 252), and in medical publications: “Gallagher said she follows the WPATH standards, which require mental health evaluations, and as a result, ‘the risk of regret is incredibly low’” (Ault, 2022). These claims also make their way into the press: “the rate of regret is very low,” “the very small group of people who detransition” (Bazelon, 2022), “regret rates between 0.3 and 0.6 percent” (Turban, 2022), “very rare” (Connell-Bryan et al., 2022), and official statements (USPATH Board & WPATH Executive Committee, 2022): “Transition reversal […] is rare.”

Knowledge of accurate rates are essential for evaluating how well the protocols for commencing medical intervention identify those who are unlikely to benefit, as well as for those considering medical intervention to weigh as risk: regret is an adverse outcome, as is detransition for some (e.g., D’Angelo, 2018; Vandenbussche, 2022). The extremely low rates are based on studies with flaws which compromise the reliability of their reported rates, or refer to a population with very different characteristics from the large numbers of young people contemplating or undergoing medical intervention today. Specifically, outcomes should not be measured too early (Abramowitz, 1986; De Cuypere et al., 2006), loss to follow-up should be small (D’Angelo, 2018; Gijs & Brewaeys, 2007; Horváth, 2018; Sutcliffe et al., 2009), and measures and definitions (Gijs & Brewaeys, 2007; Sutcliffe et al., 2009) of regret or detransition should be appropriate. In addition, the reported rates should correspond to a sample which is not biased or otherwise non-representative or irrelevant for the case of interest (D’Angelo et al., 2021; Gijs & Brewaeys, 2007). These are common and longstanding concerns for studies of medical intervention for gender dysphoria (Abramowitz, 1986; Carroll, 1999). For gender surgery in particular, Carroll (1999) identified 12 outcome study limitations over 20 years ago, most of which fall within the four requirements above (the lack of control groups and randomized treatment groups which he flags might be argued to limit the utility of these rates for determining appropriate treatment, rather than limiting the measurement of the rates themselves).

The first section below elaborates on these requirements. Next, to illustrate how the unreliability of a study can be identified quickly by checking follow-up time and loss to follow-up, studies in a recent review which provide percentages who regret surgery are examined: all but one is shown to fall short for one or both of these checks, and the last study falls short as well if a reasonable assumption is made. Then the studies giving the incorrect rates quoted above are also shown to fail one or more of these requirements, as are some other recent studies which produce a range of rates, including much higher ones. The claim here is not that the true discontinuation, detransition, or regret rates are high or low, simply that they are not known.

## Some Regret and Detransition Study Requirements

Ideally, to measure discontinuation, detransition, and/or regret, a clinic or study would wait the appropriate follow-up time and then collect discontinuation, detransition, and/or regret data from everyone who started medical transition in a well-characterized sample. The study would use accurate measurement instruments for clearly defined definitions of discontinuation, detransition, and/or regret, and, to be relevant for assessing protocols or risk, the sample would also be comparable to the sample of interest. In more detail, a study should:

### Wait Long Enough to Observe Regrets

If a study measures regret, detransition, or discontinuation outcomes prematurely, it will only provide a lower bound, as it is likely some will have not yet reached their regret, detransition, or discontinuation. Some interventions have been seen to sometimes have a honeymoon period of about a year (De Cuypere et al., 2006; Wierckx et al., 2011). Observed average or median times to regret or detransition (for different samples, interventions, measures of regret or detransition, as noted) include those for the four studies in Table 1, listed chronologically. The two regret studies listed include either a large percentage of those who had gonadectomies in the Netherlands (Wiepjes et al., 2018) or all those still living who had genital surgeries in Sweden (Dhejne et al., 2014), finding small numbers of patients (14 and 15, of 2627 and 681, respectively) who qualified as regretters with their criteria. The other two studies are convenience samples of detransitioners only, 237 in Vandenbussche (2022) and 100 in Littman (2021), with a range of interventions (31% in Vandenbussche, 2022, only socially transitioned). The first two studies concern genital surgeries of adults, and the average ages of medical transition in the second two studies are over 18, but all appear to include some who started medical intervention under age 18. The median or average time to detransition or regret range from 3.2 years in Littman (2021) for female-to-male, for a range of medical interventions, to an average of 130 months for gonadectomy (Wiepjes et al., 2018). The average and median regret times in Dhejne et al. (2014) and Wiepjes et al. (2018), where all had genital surgeries of some kind, were longer than the average detransition times in Littman (2021) and Vandenbussche (2022), where different interventions were considered. The studies in Table 1 all point to long times to reach even half of the regrets or the average time to detransition, but are at best rough estimates, as the surgical regret samples are small (Dhejne et al., 2014; Wiepjes et al., 2018), and the interventions for the much larger samples of detransitioners are heterogeneous (Littman, 2021; Vandenbussche, 2022). The long observed times to discontinuation, detransition, and regret contribute to the difficulties in obtaining adequate follow-up, discussed below.

**Table 1:** Some studies sampling regret and detransition times

Study	Quantity measured Time until regret/detransition	#(N) Regret or detransitioned	Definition of regret or detransition/ how measured	Intervention/criteria for intervention	Age (sex) when treated Total # people treated
Dhejne et al. (2014)	Median to regret 8 (all) 7.5 (FTM^a^) 8.5 (MTF^b^)	15: 5 FTM 10 MTF Median age applied 22 FTM 35 MTF	Apply for new legal sex/record search	Surgery/2 year evaluation, sterilization before starting	Median ages when all treated applied: 27 (FTM) 32 (MTF) Total treated: 252 FTM 429 MTF
Wiepjes et al. (2018)	Median to regret 73.5 months after gonadectomy, 96.5 months after starting hormones (from Wiepjes et al. 2018, Table 4) Average to regret 130 months after starting hormones	14: 3 FTM 11 MTF Median age started hormones 31 FTM 38 MTF	Reverse hormones and expressing regret in visit/ record search	Gonadectomy/screening and a diagnostic phase	Median age first visit (for adults presenting): 25 (FTM) 33 (MTF) Total with surgery: 885 FTM 1742 MTF with hormones for at least 1.5 years
Littman (2021)	Average to detransition 3.2 years FTM (2.7 SD) 5.4 years MTF (6.1 SD)	100: 69 FTM 31 MTF	Discontinued medication, had reversal surgery, or both/online survey	Medication and/or surgery/criteria to start unspecified	Ave age sought medical care: 20 FTM (SD 4.2) 26 MTF (SD 7.5) Total treated unknown
Vandenbussche (2022)	Average to detransition (includes time socially transitioned) 4.55 years FTM 6.37 years MTF SD 3.55	237: 217 FTM 20 MTF	Identify as detransitioner/online survey	Social (31%) or social + medical (65%)/criteria to start unspecified	Social (medical) transition ave age: 17.42 (20.09) FTM 23.63 (26.19) MTF SD = 5.03 (5.36) 25% started medical transition under 18 Total treated unknown

^a^FTM: female-to-male

^b^MTF: male-to-female

### Have a Small Loss to Follow-up

If those who leave the study sample (who are treated but not followed up) are different from those who did not, this will bias the sample and results. For example, people who stop medical interventions may be dropped from record searches, and stopping medical intervention is potentially related to regret, detransition, and/or discontinuation. Outside of this research field, medical study rules of thumb are that losing < 5% leads to little bias, while > 20% poses “serious threats” (Dettori, 2011, p. 9); another commonly used number (Norvell, 2010) is < 15% loss, after which the study quality is considered degraded. One way (Dettori, 2011) to estimate the maximum bias from loss to follow-up is to add those lost to follow-up to both outcomes and see what changes (“worst-case scenario”): “Only when the worst case does not change the inferences derived from the results is lost to follow-up not a problem” (p. 9). For very small regret, detransition, or discontinuation rates and large loss to follow-up, the rates will change significantly in this worst-case scenario.

It should also be noted that regretters and detransitioners do not seem likely to report their status without being asked for it; in a recent convenience sample survey of detransitioners, only about a quarter told their clinician of their detransition (Littman, 2021).

### Use an Appropriate Measurement Instrument

For detransition or regret, it is important to have a clear definition, and for the corresponding measurement to be accurate (ideally using validated or standardized assessment instruments Carroll, 1999). Questioning about regret or detransition may need to be done with care, given the range of ways regret or similar outcomes (e.g., believing medical intervention was a mistake) may manifest, and possible reluctance in reporting such outcomes: detransitioners report “a lot of negative experiences coming from medical and mental health systems and from the LGBT + community” (Vandenbussche, 2022, p. 1602) and have also reported blaming themselves (Ghorayshi, 2022). Another possible limitation is having outcomes being evaluated by the same clinicians who approved or oversaw the interventions (Carroll, 1999), which might inhibit frank responses or otherwise introduce bias. In addition, if studies do not ask about detransition or regret directly, it is possible that what is used to measure detransition or regret may not actually do so. Inadequate regret proxies include requiring a request for a name change or request for hormone reversal (some regretters may believe these will not help their situation), or counting only those who mention regret explicitly and/or spontaneously in a clinic visit or other encounter. Similarly, counting the number of people who go from puberty blockers to hormones (e.g., as described in Rosenthal, 2021) does not measure how many people regret or discontinue medical intervention. Discontinuing hormones is clearly defined; however, if people are not asked directly, the measurement method still requires care. For example, if discontinuation is measured by a search for prescription renewals, studies can either utilize several databases (van der Loos et al., 2022) and/or provide reasons that they expect the database they use is likely to be complete (Roberts et al., 2022).

### Study a Relevant Sample

If regret is measured for a sample which is too different from the group of interest, the regret rate found will not be relevant. For sample comparison, the screening protocol for starting treatment is important, as are other characteristics of the group in the study, such as age at presentation for care, age of onset, and comorbidities.

A wide variety of protocols for starting medical interventions for young people have been used, relevant for any detransition, regret, or discontinuation studies in which they might participate. The paradigmatic Dutch protocol criteria require lifelong extreme gender dysphoria, psychological stability, and family support (Biggs, 2023; de Vries & Cohen-Kettenis, 2012; de Vries et al., 2014; Delemarre-Van De Waal & Cohen-Kettenis, 2006), and treatment includes mental health support. A variation of the Dutch protocol was in use in the UK (Carmichael et al., 2021) until recently. Those medicalized with the WPATH SOC7 criteria (Coleman et al., 2012. p. 19), in force until fall 2022, had a requirement of “a long-lasting and intense pattern of gender nonconformity or gender dysphoria (whether suppressed or expressed)” for starting puberty blockers. Mental health or other issues were checked to see if they could “compromise treatment adherence” (Coleman et al., 2012, p. 19), while before treating adults with hormones, mental health concerns were recommended to be “reasonably controlled” (Coleman et al., 2012, p. 104). Many US clinicians have adopted the affirmative model (Hidalgo et al., 2013) following the American Academy of Pediatrics recommendations (Rafferty et al., 2018), which emphasizes providing the young person with the intervention they wish and which assumes that mental health issues are likely due to “minority stress” (e.g., stigma, prejudice against transgender people, etc.). For young people in the USA at or over 18, hormones are available even without a mental health evaluation (called “informed consent”; see, e.g., Cavanaugh et al., 2016).

Some current medical intervention protocols, especially for those under 18, are in flux, contributing even more potential heterogeneity to future regret, detransition, or discontinuation studies. For minors, Finland now only provides medical treatment on a case-by-case basis, prioritizing psychotherapy (PALKO/COHERE Finland, 2020), while Sweden now only recommends medical intervention in “exceptional” cases (Swedish National Board of Health & Welfare, 2022, p. 3), and the UK is turning to a more holistic approach in its proposed guidelines (NHS England, 2022). WPATH, at least for adolescents and young adults, now emphasizes more assessment and recommends that conditions which could negatively impact the outcome have been “assessed, with risks and benefits discussed” and that “other possible causes of apparent gender incongruence have been identified and excluded” (Coleman et al., 2022, p. S32; however, how to reliably accomplish the latter is not established; see Levine et al., 2022).

Outcomes for those selected and treated with one protocol might not be relevant for a sample which would not meet that protocol’s requirements. For example, the percentage of regretters who medicalized in mature adulthood after a several year screening process cannot be assumed to be indicative of likely medical outcomes for an affirmed (rather than screened) young person with adolescent onset and comorbid mental health issues. The poorly understood latter presentation (adolescent onset, often with comorbidities) dominates the current surge in cases. Also, selecting on characteristics that cannot be measured ahead of time, e.g., regret or detransition studies including only those who continue to come to a gender clinic (Davies et al., 2019; Wiepjes et al., 2018) or those who still identify as transgender or non-binary (Turban et al., 2021) misses entire classes of regretters or detransitioners who do not fall into those categories (those who stop treatment without announcing regret to their clinic and those who no longer consider themselves transgender or non-binary, respectively).

Many studies or meta-analyses of regret also combine different interventions and samples (e.g., male-to-female and female-to-male, different surgical procedures, surgeries versus only hormones, different criteria to start medical intervention, or different presentations). More specific samples would be helpful, as already noted by Carroll (1999), especially if trends in one group differ from the other (e.g., observed average regret times seem to differ between sexes).

The above flaws can diminish the reliability of other medical intervention outcome studies as well. For example, it is important to distinguish between success in attaining desired physical outcomes of hormonal or surgical interventions and the effects on gender dysphoria, mental health, and quality of life which the hormones or surgery aimed to address (Chew et al., 2018). Additional shortcomings can arise as well, for instance, studies might only find correlation, not causation, or might not account for confounding factors (e.g., mental health outcomes for medical transition may be confounded by concurrent mental health support and/or psychiatric medication). Observed regret times for a given intervention might indicate appropriate times to wait before assessing for mental health benefit for that intervention.

## Example: Follow-up Time and Loss to Follow-up Alone

**Fig. 1:**
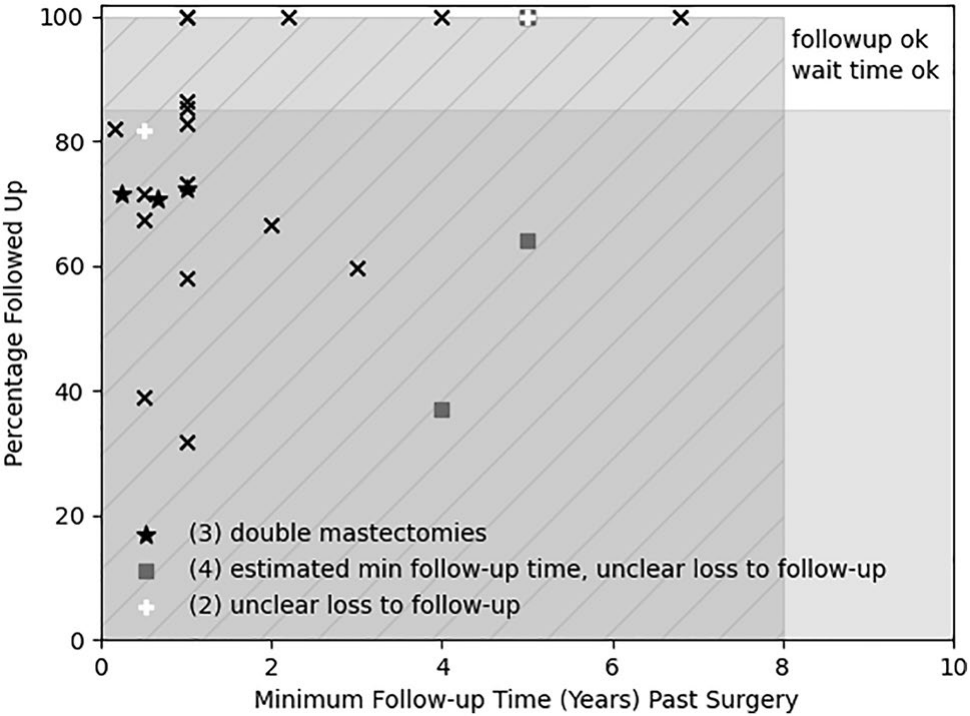
Minimum follow-up time vs. percentage followed up in Bustos et al. (2021). Studies in Bustos et al. (2021) are plotted as “x” with their minimum follow-up time (if there was a range) and loss to follow-up, except for double mastectomies (asterisks; Nelson et al., 2009; Olson-Kennedy et al., 2018; Poudrier et al., 2019), unclear loss to follow-up (white “+”: Song et al., 2011, Zavlin et al., 2018), and estimated minimum follow-up time and unclear loss to follow-up (“◾”: Judge et al., 2014, van de Grift et al. 2018, Vujovic et al., 2009, Wiepjes et al., 2018); see Appendix for details (Alleva, 2023 also discusses follow-up times). There are three studies with unknown follow-up which are placed at 100% and overlap, as they all have reported or estimated minimum follow-up time of five years or less. Loss to follow-up of more than 15% is grayed out, minimum follow-up time of less than the median regret from Dhejne et al. (2014) of ~ 8 years is both grayed out and has lines. Only studies in the white area have both sufficient follow-up time given median surgical regret times from Table 1 (at least 8 years) and sufficient follow-up (at least 85%); there are none

Many regret and detransition studies can be readily seen to produce unreliable rates just by considering follow-up time and loss to follow-up. With a conservative choice of 8 years minimum follow-up time for surgery (Table 1), all the included studies in a recent meta-analysis of surgery regret by Bustos et al. (2021) are inadequate due to excessive loss to follow-up, too short follow-up time for some patients, or both (Vujovic et al., 2009 require an additional reasonable assumption for this claim); see Appendix and Alleva (2023) for further details. One can see the numbers at a glance in Fig. 1, with the minimum time followed up past surgery for each study in the horizontal direction and the percentage followed up in the vertical direction. Studies are denoted by an “x” unless they only included double mastectomies (3 asterisks), had unclear loss to follow-up only (2 white “+” signs), or had an estimated rather than reported minimum follow-up time in addition to unclear loss to follow-up (4 filled squares; one study, Vujovic et al., 2009, also involves the assumption that some had surgery from those presenting in the last time period they studied); the follow-up percentages shown for these last two sets of studies either give other information or are placed at 100% to indicate they are unknown. More details are given in Appendix and Table 3. The white area in the upper right-hand corner is where studies meeting both > 85% follow-up and over 8 years follow-up for all patients would lie; there are none. (These studies, in fact, all have minimum follow-up time less than 8 years.) It is possible that double mastectomies might have a different characteristic regret time than genital surgery; however, the regret studies for double mastectomies only (Nelson et al., 2009; Olson-Kennedy et al., 2018; Poudrier et al., 2019) included participants followed up for one year or less, an even shorter time than the shortest observed average detransition or regret time in Table 1 (3.2 years in Littman, 2021).

## More Detailed Examples: Some Frequently Quoted Studies and Their Shortcomings or Restrictions

There are several other studies frequently quoted for low regret rates besides Bustos et al. (2021). Table 2 lists follow-up time, loss to follow-up, definition and measurement, and sample characteristics for several regret, detransition, and discontinuation studies, for comparison with the four study criteria discussed above. The studies whose numbers are quoted in the introduction are indicated by asterisks. In addition, a selection of other studies are also included as examples: three studies cited by GenderGP (2021; GenderGP is an online company which provides medication prescriptions for young people internationally and claims a 3% regret rate for medical intervention), as well as six recent studies, three showing larger rates (e.g., Roberts et al., 2022, found almost 30% discontinued hormones in four years) and three showing very small rates. Three of these six recent studies include a large percentage of people who were under 18 when starting medical intervention (Roberts et al., 2022; Tang et al., 2022; van der Loos et al., 2022). The requirement(s) which each study does not meet is/are listed in the second column of Table 2. For a few studies, the main problem is too short a minimum follow-up time. However, some studies are particularly hard to interpret; for instance, Narayan et al. (2021) sent a survey to a group of surgeons asking how many encounters they had had where a patient expressed regret; the regret rate was then calculated to be the number of such encounters divided by the total number of surgeries done by those surgeons. This method has unclear follow-up time, loss to follow-up, and even measurement instrument (were questions about regret routinely asked and was there a consistent definition of regret in use?). Similarly, the regret rate of Jedrzejewski et al. (2023) includes only detransitioners who were “recorded” and patients who requested reversal surgery.

**Table 2:** Flaws and limitations of several regret, detransition, and discontinuation studies

Study outcome & rate	Possible limitations	Follow-up time	Loss to follow-up (LTFO)	Defn/how measured	Sample	Intervention/criteria	Ages, notes
Dhejne et al. (2014) Regret 2.2% (4.4% if require 10 yr follow-up, i.e., 1960–2000)	Short min follow-up time, Definition of regret, Sample: older with different criteria for starting	0–50 years	From 1973 to 2003 8.3% died^a^, (Full study sample 1960–2010)	Apply for new legal sex/Record search	252 FTM and 429 MTF living adults in Sweden who had applied for and received surgery + legal change	Full surgery/2 year evaluation, sterilization before starting	Median ages for all & for regretters when applied: 27 & 22 (FTM) 32 & 35 (MTF) At least 18 for new legal gender, minimum application 16 years old
*Wiepjes et al. (2018) Regret < 1%	Short min follow-up time, Unclear LTFO, Definition of regret, Search method	Some ≤ 5 years (see Appendix for details of estimate)	36% of everyone at clinic	Reverse hormones and express regret in visit/record search	885 FTM and 1742 MTF who had surgery, treated with hormones at least 1.5 yrs, and who had not dropped out of treatment	Gonadectomy/screening and diagnostic phase	At least 18
Davies et al. (2019) Detransition 0.47%	Unknown follow-up time, Unknown LTFO, Search method, Sample: active clinic patients	Unknown	Unknown	Records in 1 year period/Electronic record scan for words related to detransition or regret plus review by authors	3398 active patients coming to gender clinic in 1 year period	All people in treatment at adult clinic/criteria unknown	Adults (adult clinic)
Hall et al. (2021) Detransition 6.9%, another 3.4% pattern suggesting detransition	Short follow-up time	16 months after discharge	3/175 unclear or transfer	Reverting to original role/retrospective case note review	Natal female: 59 FTM, 8 NB Natal male: 101 MTF, 6 NB, 1 to detransition 175 discharged patients from single national clinic w/in 1 year who did not transfer to another clinic	Mixed/2 assessments	17 and over/1.7% suicides, 2.3% detrans then re-referred Median age at discharge: 20 FTM or NB 36 MTF or NB
*Narayan et al. (2021) Regret 0.2–0.3%	Unknown follow-up time, Unknown LTFO, Regret measure, Sample: encounters	Unknown	Unknown	Express regret in encounter with surgeon/ask surgeons in survey about number of regret encounters	Between 18,125 and 27,325 patients which responding surgeons had operated on (regret encounters did not have to be former patient)	Surgery/criteria mixed	Ages unknown/Rate is regret encounters/Total patients w/surgeries by responding surgeons 62 regretters
Turban et al. (2021) Detransition 13.1% all, 9.1% hormones, 6.8% surgery	Unknown follow-up time, Unknown LTFO, Sample: both required to still be TGNB and non- representative^b^	Unknown	Unknown	Self-described detransition/online convenience survey	6289 FTM 7191 MTF 3671 other pursued gender affirmation and still identify as trans or non-binary	Social, hormones, and/or surgery/criteria unknown	11% under 18 when first started living as other gender, > 18 at time of study
Boyd et al. (2022) Discontinue 20% Detransition 10% Regrets but trans 2.5%	Unclear follow-up time	Unknown	N/A current patients	Discontinue hormones plus eval records/NHS medical records	41 on hormones in GP practice population at regional clinic	Hormones or surgery/Some “affirmed”	Adults who detransitioned took testosterone for a mean of 18 months, presented at median age of 18/“not generalizable”
Roberts et al., 2022 Discontinue ~ 30%	Short follow-up time	4 years	N/A	Discontinue hormones/Prescription renewals plus still in medical system	627 FTM and 325 MTF who were still eligible for treatment within medical system and still went to it for other medical care	Hormones/criteria unknown	39.1% under 18 when started
Tang et al. (2022) Regret 2/209	Short min follow-up time, Search method, Outcome measure	Some < 1 year	17/209 outcome not documented, 72/209 (34%) do not have at least 1 year follow-up in records	Regret mentioned to surgeon or MHP in system notes/record search	209 patients still in medical system	Double mastectomy/Therapist assessment, surgeon informed consent, real life test for a year preferred	double mastectomy at ages 12–17, all FTM/Regretters at 11 months and 1.5 years
van der Loos et al. (2022) Discontinue 2%	Short min follow-up time	Min, median time since started FTM 0, 2.3 yr MTF 0.1, 3.5 yr	Appears to be 5/720, 3 of whom died	Discontinue prescription/Record search clinic plus insurance	500 FTM 220 MTF	3 month GnRHa and hormones/referred by physician and diagnosed with gender dysphoria	All < 18 when started GnRHa, many became young adults by study end 0.4% died
Jedrzejewski et al. (2023) Regret 0.3%	Short min follow-up time, Unknown LTFO, Regret measure, Sample: encounters	0 (to 1.4) to < 5.6 (to 7) years^c^	Unknown	Request for reversal surgery or encounter of clinic with detransitioner/Encounters at clinic	All 1989 patients operated on from Jan. 1, 2016 to July 31, 2021	Surgery: varied, e.g., chest feminization or masculinization, orchiectomy, hysterectomy	Initial consult mean age 24.5 years old (16–37) Rate: reversal requests or encounter with detransitioner/ total surgeries in same period 6 regretters

*Study quoted in introduction as example of low regret or detransition rate

^a^Dhejne et al. (2011)

^b^D’Angelo et al. (2021)

^c^The regretters had their surgery in the time period January 2016–July 2021. It is not clear when follow-up ended, in principle it could have been any time from July 2021 to December 2022, the month before the paper appeared in January, 2023

## Discussion

Regret and sometimes detransition are negative outcomes. Loss to follow-up, premature outcome measurement, flawed instruments, and irrelevant samples all contribute to produce unreliable regret, detransition, or discontinuation rates. The proposed follow-up of NHS GIDS Tavistock outcomes (Crawford, 2022) shows promise, as do the proposed requirements for research contexts for new minor patients in Sweden (Swedish National Board of Health & Welfare, 2022). Research settings have also been proposed for puberty blockers for new minor patients by NHS England (2022), with similar proposals in preparation regarding hormones. There is also a two year follow-up time for a US-funded, four-center study underway (Chen et al., 2023; Olson-Kennedy et al., 2019); however, this appears too short to reliably measure long-term outcomes given the average observed regret and detransition times, albeit for older populations, as shown in Table 1. Better follow-up studies can help better constrain regret, detransition, and discontinuation rates, and other significant unknowns as well, such as the rates and time scales for detransition followed by retransition (Turban et al., 2021) and the rates of positive long-term outcomes. Although there are many anecdotal reports of positive long-term outcomes, inadequate long-term follow-up limits understanding how common these are as well.

These outcome rates alone cannot illuminate how well medical intervention itself helps gender dysphoria, as the “no treatment” alternative, the natural history of gender dysphoria, is not well understood, especially for those with adolescent onset (Kaltiala-Heino et al., 2018). In particular, it is unknown how to reliably determine when any given individual’s gender dysphoria might be temporary (Levine et al., 2022). Some temporary cases of gender dysphoria have been associated with the presence of comorbidities, including autism spectrum disorders, obsessive compulsive disorders, separation anxiety, depression, anorexia, homophobia, or trauma (Bockting et al., 2006; Churcher-Clarke & Spiliadis, 2019; Evans & Evans, 2021; Parkinson, 2014; Withers, 2020), conditions frequently present in the current large adolescent cohort (Kaltiala-Heino et al., 2018). Exploratory psychotherapy (Ayad et al., 2022; Evans & Evans, 2021), group therapy (Withers, 2020), and other supportive mental health interventions sometimes resolve gender dysphoria without medical intervention. These alternatives to medical intervention are also currently backed by low-quality evidence (Levine et al., 2022). Evidence would benefit from “randomized controlled trials, well-conducted comparative observational studies or very large case series (which include a large sample of consecutive patients who are representative of the whole population)” (Brignardello-Petersen & Wiercioch, 2022, p. 5).

One can compare the state of research regarding gender dysphoria with what is known about depression, also an increasingly prevalent condition for adolescents (Haidt & Twenge, ongoing), also with several different forms, and also with both medical and mental health interventions. For adolescents, the (439 participant multicenter) Treatment for Adolescents with Depression Study randomized controlled trial found combining psychotherapy and pharmacology was optimal for moderate to severe major depressive disorder, compared to one or the other (or a placebo for the pharmacological arm alone), and associated with persistence of benefits one year after treatment discontinuation (March et al., 2007; March & Vitiello, 2009; Treatment for Adolescents With Depression Study [TADS] Team, 2009). No such study has been done for gender dysphoria. High-quality evidence supports use of antidepressants and moderate quality evidence supports use of cognitive behavioral therapy (mental health intervention) for major depressive disorder (Gartlehner et al., 2017), while moderate or high-quality evidence for medical intervention for gender dysphoria is not yet available. A large percentage of systematic reviews of depression treatments have critically low-quality methodology, in particular those which are not Cochrane reviews (Matthias et al., 2020), but there are over 100 Cochrane systematic reviews (Cochrane Database of Systematic Reviews, 2022) for various possible treatments for depressive disorders, compared to only three Cochrane reviews with the words gender dysphoria or transgender in their title, abstract or keywords. As far as outcome statistics, the percentages of times different interventions for depression have succeeded or failed (according to specific measures) have been compiled using meta-analyses (Cuijpers et al., 2020; some outcome statistics for adolescents were already reported in March et al., 2007), while for gender dysphoria, reliable regret, detransition, and discontinuation rates, and other outcome statistics, are unknown. There are still many questions, for instance, recurrent major depressive disorder is perhaps the most analogous to gender dysphoria in its need for longer-term studies (Severe et al., 2020), and most depression studies do not address longer-term effects (Cuijpers et al., 2020); however, the research on depression illustrates that much better studies are possible, even if a condition is complex, involves mental health symptoms, and has both medical and psychological interventions.

Extending the comparison to depression beyond studies, treatments for depression have a known placebo effect and medical interventions for gender dysphoria are now postulated to have one as well (Clayton, 2023). That is, components of the treatment (or research) context, such as expectations of positive benefit, positive clinician–patient interaction, and/or community influence (social observational learning), might stimulate beneficial psychobiological responses, rather than the medical intervention itself doing so. Psychotherapy is largely well tolerated for both depression and gender dysphoria. In contrast, the physical consequences of medical intervention for gender dysphoria are profoundly different from their counterparts for depression, which usually have no long-term adverse physical effects.

In addition, although failure to resolve depression with medical treatment is an unwanted outcome, especially given the dangers of depression, when someone regrets, detransitions, or discontinues medical intervention for gender dysphoria, serious consequences from medical intervention frequently remain, often for life. These can include sterility (Cheng et al., 2019), loss of breasts, irreversibly harmed genitals and/or sexual function, and lifetime dependence upon medication due to gonad removal, as well as loss or growth of hair, voice changes, and breast growth. Detransition can also incur detrimental social consequences: many in the Vandenbussche (2022) study “expressed a feeling of rejection and loss of support in relation to their decision to detransition, which lead (sic) them to step away from LGBT + groups and communities” (p. 1611). That is, not only might their gender dysphoria not be resolved, but the medical harm they incurred in order to try to resolve it and the consequences of detransition or feeling regret may create additional, sometimes devastating, challenges. In one convenience sample of detransitioners who identified as such, 60% reported needing help in learning to cope with feelings of regret (Vandenbussche, 2022). In another convenience sample, comprised of those who medically detransitioned, almost two-thirds “indicated that if they knew then what they know now, they would not have chosen to transition” (Littman, 2021, p. 3364). Negative outcomes can be extremely painful, and cause long-term suffering: “Some of us will now never be able to have children and many of us live with great distress and regret every day” (Fox, 2021). Knowing the number of those who discontinue, detransition, and/or regret is clinically crucial, but currently unavailable information.

Given the long-known and well-known limitations of outcome studies, one might question why these issues have not been addressed sooner. Medical interventions for gender dysphoria are already widespread: in the USA alone, over 17,000 children aged 6–17 started puberty blockers or hormones from 2017 to 2021, with at least 56 genital surgeries and 776 double mastectomies in the 13–17 age range from 2019 to 2021 (Respaut & Terhune, 2022). In response to the lack of systematic outcome studies, the irreversible nature of many of the hormonal and surgical interventions, and the serious consequences of poor outcomes, it has recently been asked (Clayton, 2022, p. 695): “Why are these experimental interventions, with inherent risks and scarce, low-quality evidence for benefits, being implemented outside HREC regulated clinical trial settings?”.

It is important for those considering medical intervention to know that the likelihood of regret, detransition, and discontinuation is unknown, that regret and detransition can be traumatic, and that the extremely small numbers quoted by some (Bustos et al., 2021; McNamara et al., 2022; Rosenthal, 2021; Turban, 2022) are not reliable or representative. With patient preferences for treatment pathways paramount in the common affirmative model, it is crucial that risks and benefits of medical intervention, and those of the alternatives, be clearly communicated, including accurate information about detransition and regret rates. Until more is known, young people considering these interventions and their families should be made aware of this significant uncertainty as part of informed consent, following the recommendations of Levine et al. (2022).

## Appendix

Table

**Table 3:** Shortest surgery follow-up time in study and percentage followed up for studies in Bustos et al. (2021)

Authors, Symbol in Fig. 1 if not “x”	Shortest follow-up time past surgery	Fraction or percentage followed up	#FTM	#MTF
Blanchard et al. (1989)	1 year	111/134^a^	61	50
Bouman (1988)	2 months	55/67		55
Cohen-Kettenis and van Goozen (1997)	1 year	19/22	14	5
De Cuypere et al. (2006)	1 year	62/107	27	35
Garcia et al. (2014)	Mean 6.8 years Mean 2.2 years	15/15 10/10	25	
Imbimbo et al. (2009)	1 year	139/163		139
Johansson et al. (2010)	2 years	32^b^/48; 14 FTM, 18 MTF	14	18
Judge et al. (2014), “■”	≤ 5 years (see Appendix)	shown as 1 55 reported to have had surgery	19	36
Krege et al. (2001)	6 months	31/46		31
Landén et al. (1998)	4 years	213/213	93^c^	120^c^
Lawrence (2003)	1 year	232/727		232
Lobato et al. (2006)	1 year	19/26	1	18
Nelson et al. (2009), “*”	8 months	12/17	12	
Olson-Kennedy et al. (2018), “*”	Some < 1 year	68/94 [authors also say 68 respondents, 26 lost to follow-up and 93 total]	68	
Papadopulos et al. (2017)	6 months	47/121		47
Pfäfflin (1992) Sample 2	1 year	295/295	99	196
Poudrier et al. (2019), “*”	3 months	58/81	58	
Rehman et al. (1999)	3 years	28/47		28
Smith et al. (2001)	1 year	20/20	13	7
Song et al. (2011), white “+”	5 years	Shown as 1 8/19 reported had 5 year follow-up time, unclear if others also had 5 yrs	8	
van de Grift et al. (2018), “■”	< 4 years (4 years after first clinical contact)	37% (for all interventions)	51^d^	81^d^
Vujovic et al. (2009), “■”	≤ 5 years (assumption made, see Appendix)	Shown as 1 118 reported to have had surgery	59	59
Weyers et al. (2009)	6 months	50/70		50
Wiepjes et al. (2018), “■”	≤ 5 years (see Appendix)	Shown as 64% hormones, not necessarily surgery	885	1742
Zavlin et al. (2018), white “+”	6 months	Shown as 40/49 49 agreed to participate, 40 did		40

^a^134 patients had surgery, but 2 died before the study, so Blanchard et al. (1998) take the full sample as 132 people

^b^One of the 48 died from surgical complications

^c^Table 1, Landén et al. (1998), subtracting 1 FTM and 4 MTF who were added to calculate regret characteristics but not frequency

^d^A total of 136 respondents had surgery, however, 4 of them had data missing, see van de Grift et al. (2018), Fig. 1

^3^ lists the studies from Bustos et al. (2021) which appear in Fig. 1, with their corresponding symbol in Fig. 1, their shortest included follow-up time, the percentage followed up (see also Alleva, 2023; Expósito-Campos & D’Angelo, 2021), and breakdown in terms of female-to-male (FTM) and male-to-female (MTF). Further details about the individual studies are available in Bustos et al. (2021), Table 2.

Most of the Bustos et al. (2021) studies are shown as “x” in Fig. 1. The three studies which only include double mastectomies are denoted by asterisks (Nelson et al., 2009; Olson-Kennedy et al., 2018; Poudrier et al., 2019).

For four studies, shown as filled squares, the minimum follow-up time can be estimated to be below some number of years (the follow-up percentage is unclear for these as well, but the too small minimum follow-up time is sufficient to question the studies’ results for regret). An additional (reasonable) assumption is needed for Vujovic et al. (2009). van de Grift et al. (2018) followed up 4 to 6 years after first clinical contact, so the study’s minimum follow-up time is no larger than 4 years. For Wiepjes et al. (2018), records were searched in 2015, and a large fraction of the patients who first came to the clinic in 2010 were seen to have had surgery by 2015 (Wiepjes et al., 2018, Fig. 2, bottom); they thus had surgical follow-up of 5 years at most. For Judge et al. (2014), 52 of the patients in the study had surgery after they were referred, and only 38 study patients were referred in 2008 or before (Judge et al., 2014, Table 2), so at least 14 of the patients referred in 2009 or later had surgery. As the study used records from 2014, those with surgery in 2009 or later were followed up for 5 or fewer years.

For Vujovic et al. (2009), an additional assumption is needed, as their Fig. 1 which reports the years of presentation of their 147 patients shows numbers per three-year period which add up to over 180 patients (they report a loss to follow-up only for the 147). If any of those who presented in their last 3-year period (2003–2006) went on to surgery, their protocol of having at least a year of hormones would have resulted in surgery in 2004 or later. As the study was published in 2009, such patients would thus have had follow-up time of 5 years or less. The assumption is that some patients in the group presenting from 2003 to 2006 had surgery. This is not unreasonable, as over 75% of all of the 147 the study discusses had surgery within two years of staring hormones, and so, it would be expected that the study authors would have mentioned if none had done so from the large number presenting in their last studied time period (comprising almost 1/3 of the total presenting over all 5 time periods). The follow-up percentage for all four of these studies is also not clear, but not as important, given these small estimated follow-up times, so the numbers which are displayed provide other information (the 37% follow-up percentage in van de Grift et al., 2018 uses as the denominator all those presenting for treatment in the study period, and the 64% follow-up percentage from Wiepjes et al., 2018 refers to all those on hormones, regardless of surgical intervention) or no information (Judge et al., 2014 and Vujovic et al., 2009 are placed at 100% follow-up percentage and lie at the same point in Fig. 1).

Shown by white “+” signs, Song et al. (2011) and Zavlin et al. (2018) give follow-up time but unclear follow-up percentage; Song et al. (2011) is displayed at 100%, Zavlin et al. (2018) report that 49 agreed to participate in the study but that only 40 did, so the displayed percentage corresponds to 40/49. The three studies (Judge et al., 2014; Song et al., 2011; Vujovic et al., 2009) overlap at 100% follow-up fraction placement and minimum follow-up time being no larger than 5 years.

Two studies from Bustos et al. (2021) are dropped from Fig. 1 and Table 1: Jiang et al. (2018) studied regret for which kind of surgery was chosen, and Kuiper and Cohen-Kettenis (1998) did not find a regret percentage, as they only sought regretters.
